# Confinement induced change of microemulsion phase structure in controlled pore glass (CPG) monoliths†

**DOI:** 10.1039/d4ra04090b

**Published:** 2024-09-04

**Authors:** Margarethe Dahl, Cedric J. Gommes, René Haverkamp, Kathleen Wood, Sylvain Prévost, Pierre Schröer, Tomáš Omasta, Tim Julian Stank, Thomas Hellweg, Stefan Wellert

**Affiliations:** a Department of Chemistry, Technische Universität Berlin Straße des 17. Juni 124 10623 Berlin Germany s.wellert@tu-berlin.de; b Department of Chemical Engineering, University of Liège B6 A 3 Allée du 6 Août B-4000 Liège Belgium; c Department of Physical and Biophysical Chemistry, University of Bielefeld, Universitätsstraße 25 33615 Bielefeld Germany; d Australian Nuclear and Technology Organisation New Illawarra Rd Lucas Heights NSW 2234 Australia; e Institut Laue-Langevin 71 Avenue des Martyrs F-38042 Grenoble France

## Abstract

We use small-angle neutron scattering (SANS) to investigate the structure and phase behavior of a complex fluid within meso- and macroporous matrices. Specifically, bicontinuous microemulsions of the temperature-dependent ternary system C_10_E_4_–water–*n*-octane are investigated in controlled pore glass (CPG) membranes with nominal pore diameters of 10 nm, 20 nm, 50 nm, and 100 nm. The scattering data were analyzed using the Teubner–Strey model and a multiphase generalization of clipped Gaussian-field models. The analysis indicates changes in the phase structure of the bicontinuous microemulsion in the membranes with the smallest pores. This is attributed to a shift in the ternary phase diagram toward a three-phase structure at lower surfactant concentrations. This effect is likely related to a larger internal surface area in the membranes with smaller pores, which enhances surfactant adsorption onto the pore walls.

## Abstract 2

We use small-angle neutron scattering (SANS) to investigate the structure and phase behavior of a complex fluid within meso- and macroporous matrices.

## Graphical abstract

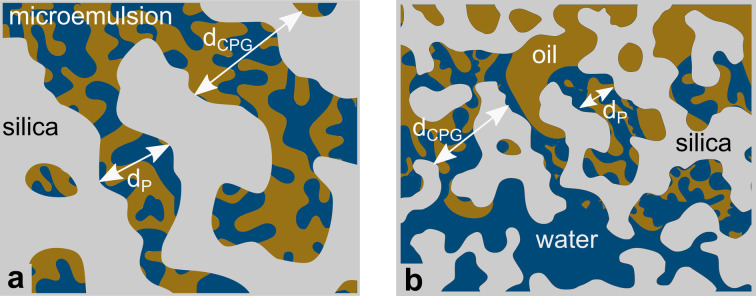

## 1 Introduction

Investigations in the field of soft matter in confinement are very interdisciplinary in nature.^1,2^ Typical length scales of soft matter are in the range of nanometers and micrometers, and relevant thermal energies of the order of a few *k*_B_*T*. Forcing soft matter into geometric confinement can reduce its degrees of freedom, influence its phase behavior and structure, or induce changes in molecular interactions. Such systems include colloidal dispersions, polymer solutions or nanoscopically structured complex fluids inside pores or channels.^1–3^

The confinement may be static, such as porous solid materials, channels, or membranes, or dynamic, such as a crowded environment or time-variant gradients in an external field.^4^ Static confinement effects depend on several material properties of the confining matrix, like the pore size, shape, and surface area, as well as the chemical composition of the pore walls, which affects the interaction between the solid surface and the confined soft matter.^5,6^ Confinement effects in solid porous materials include the hindered formation of ice, vapor condensation, *etc.*^7,8^ Confined liquids such as binary mixtures of simple liquids can deviate strongly from their behavior in bulk, including the absence of the macroscopic phase separation, shift, and shrinkage of the miscibility window.^6^ These effects can be accompanied by preferential wetting of one of the two phases, a suppression or slowing down of macroscopic separation, and a reduction of critical fluctuations inside the pores. Different from these binary mixtures exhibiting a miscibility gap are microemulsions, which are thermodynamically stable mixtures of oil and water in the presence of surfactant molecules. Here, the solid confinement and its extremely large internal surface area might affect the phase behavior and structural properties of the confined microemulsion due to interactions of these complex fluids with the solid interface.^9–13^

Microemulsions are thermodynamically stable colloidal systems spontaneously formed by water, oil, and an amphiphile. Their unique properties are desirable for a wide range of applications,^14^ including pharmaceutics,^15^ cosmetics,^16^ enhanced oil recovery (EOR),^17,18^ decontamination^19,20^ and remediation.^21^ In addition to its fundamental interest, the understanding of the behavior of microemulsions in porous materials is central for most applications. The microemulsion utilized in this study is a ternary system based on a non-ionic surfactant belonging to the alkyl oligoethylene oxide (C_*i*_E_*j*_) class. These systems have been extensively studied^22–25^ and they provide a valuable platform for studying fundamental aspects of phase behavior in confinement. The composition of the microemulsion and temperature in this study were selected to be in the center of the bicontinuous single-phase region. The water and oil domains are separated by a surfactant monolayer, which forms a sponge-like pattern. Droplet microemulsions were already explored inside cylindrical pores and reported deformation of these droplets when the pore size was smaller than the droplet.^26^ Several studies on the near-surface structure of bicontinuous microemulsions with a planar confining surface reported the existence of surface-induced lamellar ordering.^9–13^ Recently, we discovered that the temperature-dependent behavior of the microemulsion is suppressed in small pores.^27^

This study focuses on controlled pore glasses (CPG) as model porous materials. These are disordered sponge-like silica structures with a narrow pore size distribution.^6,28^ Their structure has been studied by different methods, including electron microscopy and small angle scattering (SAS).^29,30^ SAS is a unique tool to elucidate the nanometer-scale structure of soft matter inside a porous material,^31–34^ but it relies on suitable scattering data analysis methods. Traditional approaches include Cahn's decomposition model^35^ extended by Teubner and Strey (TS),^36^ which describe well the main feature of the scattering signal but are unable to describe the signal over the complete *q*-range. Other approaches are based on multi-phase generalizations of clipped Gaussian random field models,^37,38^ which enables one to reconstruct the microemulsion nanometer-scale structure inside the mesopores.^39^

To our knowledge, this complex fluid inside a disordered porous material has not been studied by small-angle neutron scattering (SANS). This study compares two distinct methodologies for analyzing the intricate SANS signal. The first approach is a conventional fitting method, and the second is modeling the invariant *Q* and applying plurigaussian fitting. Furthermore, the influence of the surface polarity and the pore size of the confining matrix is investigated. Such insights are crucial for advancing our understanding of confined soft matter systems and improving the design of novel materials for applications based on bicontinuous microemulsions.

## 2 Experimental

### 2.1 Materials

Water was purified using a Milli-Q system (Millipore), reaching a final resistance of 18 MΩ. Tetraethylene glycol monodecyl ether (C_10_E_4_) (>95%) was purchased from Bachem. *n*-Octane, dichlorodimethylsilane (DCDMS), and anhydrous toluene (99.8%) were purchased from Sigma-Aldrich. Sulfuric acid (95%) and aqueous solution of hydrogen peroxide (30%, stabilized) were purchased from VWR Chemicals. Deuterium oxide (D_2_O, 99.9%) was purchased from Deutero. Chemicals were used as received.

Monolithic CPG membranes with dimensions 10 × 10 × 1.2 mm (*L* × *W* × *H*) were purchased from Boraglas GmbH. Pore diameter (*d*_P,Hg_), pore volume (*V*_P_), and porosity (*ε* = *V*_P_/*V*) were determined by Hg intrusion measurements from the supplier and can be found in the Table 2. The pore size distribution can be found in the ESI Fig. S1.† The CPGs were cleaned according to the following procedure. First, they were cleaned in Milli-Q water using an ultrasonic bath and dried under a nitrogen stream. Then the CPGs were immersed in a Piranha solution (v/v = 1 : 1; H_2_SO_4_, H_2_O_2aq._) for 20 min. To remove the Piranha solution from hard-to-reach pores, the CPGs were first thoroughly rinsed with water and placed in an ultrasonic bath for 15 min. Afterward, the CPGs were dried overnight in a vacuum furnace at 40 °C.

### 2.2 Surface modification of CPG membranes

The surface polarity was altered following a modified procedure of Bosley and Clayton.^40^ 25 mL Anhydrous toluene per CPG membrane was transferred into a glass reactor, purged with nitrogen for 30 min, and stirred at 300 rpm. Afterward, 7 mL DCDMS was added to the toluene and stirred for 5 min at 600 rpm. The cleaned and dried CPG membranes were placed separately in a PTFE tube. Then 25 mL of this solution was added to each PTFE tube and placed on an orbital shaker for 1 h at room temperature. The CPG membranes were subsequently washed twice with toluene, acetone, and Milli-Q water using an ultrasonic bath for 10 min and dried in a vacuum furnace at 40 °C.

### 2.3 Methods

#### 2.3.1 Microemulsion preparation and characterization

**Fig. 1:**
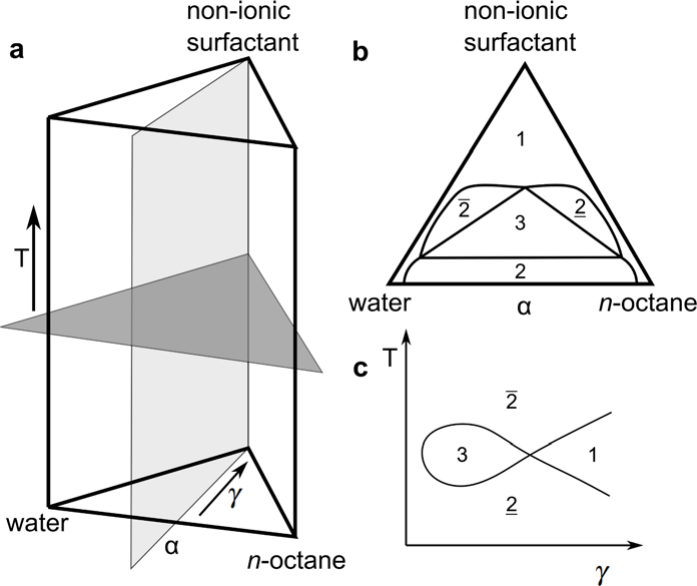
(a) Schematic representation of the three-dimensional phase prism formed by the ternary system C_10_E_4_–water–*n*-octane and temperature *T* in the vertical direction as tuning parameter for the curvature of the amphiphilic interface between water and oil. (b) With constant temperature, a two-dimensional horizontal cut through the prism is achieved, forming a ternary phase diagram. It shows the existence of single-, two-, and three-phase regions. (c) In the case of a fixed oil-to-water ratio *α* = 0.5, the phase prism is cut in the vertical direction, forming the well-known two-dimensional fish-type diagram. For further details on the phase behavior, see.^22,23,41^

The temperature-dependent phase behavior of the ternary system C_10_E_4_–H_2_O–*n*-octane is well characterized and discussed in the literature.^25,41^ The general features of temperature-dependent phase behavior in a ternary oil–water–surfactant system are shown in Fig. 1. Since the investigated system was already intensively studied, it was not necessary to fully characterize the phase behavior. All analyzed samples originate from the bicontinuous phase of the system.^25,42^ The bicontinuous microemulsion was prepared using equal volumes of *n*-octane and heavy water, corresponding to volume fraction
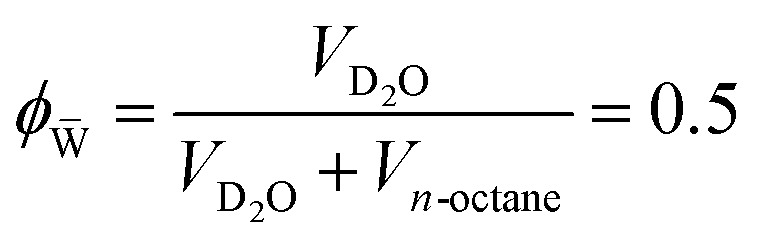
and a surfactant concentration of 12.84 wt% obtained from
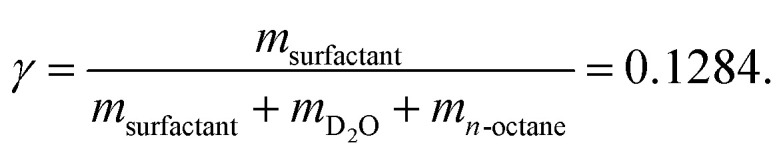
To ensure that the samples are bicontinuous and monophasic, the microemulsion samples were stored in a thermostated cabinet at *T* = 22 °C prior to all measurements.

#### 2.3.2 N_2_-porosimetry

Nitrogen adsorption/desorption isotherms were measured on an Autosorb-1 (Quantachrome) at 77 K. Before the measurements, the samples were degassed under vacuum at 90 °C for 24 h to remove any impurities. The specific surface area (*A*_S_) was obtained using the multi-point Brunauer–Emmet–Teller (BET) method.

#### 2.3.3 Surfactant adsorption onto CPG

The adsorption from aqueous C_10_E_4_ solutions onto hydrophilic and hydrophobic CPG was studied using the method of depletion.^43^ The CPG membranes were placed in the surfactant solutions for 24 h at 22 °C. After removal of the CPG, the equilibrium surfactant concentration of the supernatant was determined by measuring the surface tension with a Du-Noüy ring on a DCAT tensiometer (Data Physics) at 22 °C, using a calibration curve of the surface tension *σ* over the surfactant concentration log *c* of an aqueous C_10_E_4_ solution. For surfactant concentrations above the critical micellar concentration (cmc), the supernatant was diluted with water until the surface tension *σ* was higher than *σ*_cmc_.

**Fig. 2:**
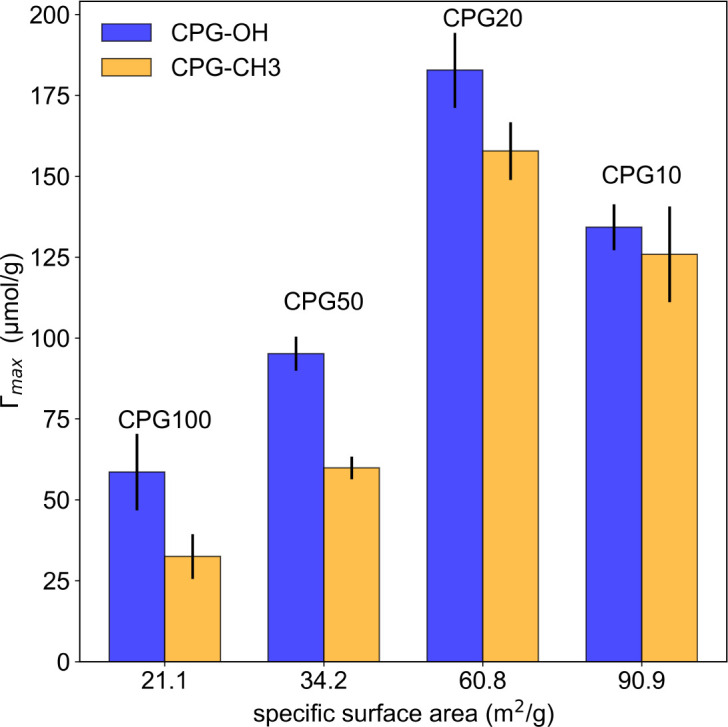
Maximum adsorbed C_10_E_4_ surfactant from aqueous solution onto hydrophilic CPG (blue) and hydrophobic (yellow).

The S-shaped isotherms are described by the equation by Gu and Zhu^44^ and can be found in the ESI in Fig. S2.†1
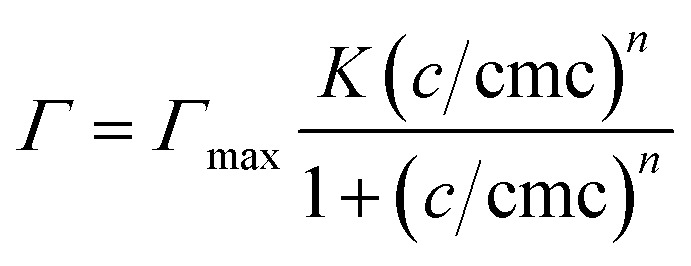
The maximum amount of adsorbed surfactant *Γ*_max_ is extracted from the isotherms and shown in Fig. 2 against the specific surface area for hydrophilic and hydrophobic CPGs.

#### 2.3.4 Small angle neutron scattering

Small angle neutron scattering (SANS) experiments were performed at the D22 instrument at the Institut Laue-Langevin (ILL, Grenoble, France)^45^ and at the Quokka instrument at the Australian Nuclear Science and Technology Organisation (ANSTO, Sydney, Australia).^46^ All data are expressed as the scattering cross-section against the magnitude of the scattering vector **q** given by 
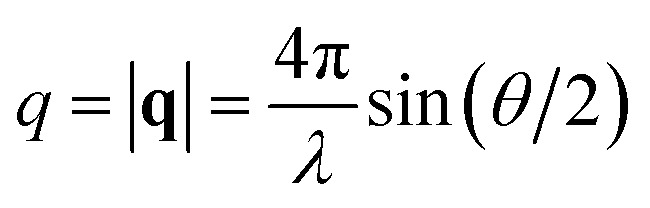
, where *θ* is the scattering angle and *λ* is the neutron wavelength.

The Quokka instrument was used with three different configurations. The wavelength (*λ*) and sample-to-detector distance (*l*_SD_) were set to *λ* = 6 Å, *l*_SD_ = 1.35 m (high *q*), *λ* = 6 Å, *l*_SD_ = 8 m (mid *q*), *λ* = 8.1 Å with a lens focusing optics, *l*_SD_ = 20 m (low *q*). With these configurations a *q*-range of 5 × 10^−4^ to 0.6 Å^−1^ was covered. At the D22 instrument a single configuration with a *λ* = 6 Å and two separate detectors at the distance of *l*_SD_ = 17.6 m and *l*_SD_ = 1.4 m with an angle of 20° was used which leads to a covered *q*-range of 2.6 × 10^−3^ to 0.64 Å^−1^.

The bicontinuous microemulsion was measured inside Hellma QS cells (path length of 1 mm). The CPG membranes were placed between two quartz windows in a sandwich cell for solids. A Viton spacer with a thickness of 1.4 mm was used as a sealing, which resulted in a path length of 1.2 mm. The required amount of microemulsion was adjusted according to the porosity and added to the CPG membrane before closing the sandwich cell with screws. All measurements were performed at 22 °C.

The data reduction at the D22 was performed using the Grasp v10.17 software, normalizing with monitor, subtracting the contribution from the empty cell, taking into account noise from the measurement with a sintered^10^B_4_C piece at the sample position, and using for transmission the intensity from the attenuated direct beam. At the Quokka instrument, the data reduction was performed using macros written in IGOR.^47^ The data were analyzed using Python and Matlab.

## 3 Scattering data analysis

The differential scattering cross-section d*Σ*/d*Ω*(*q*) per unit volume of the material is the Fourier transform of the scattering-length density correlation function. In the case of an isotropic structure, this takes the form2
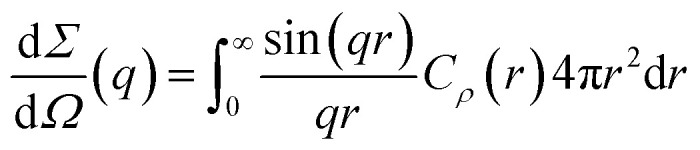
where

3 *C*_*ρ*_(*r*) = 〈*ρ*(**x**)*ρ*(**x** + **r**)〉 − 〈*ρ*〉^2^

In this equation, *ρ*(**x**) is the local scattering-length density at point **x** and the brackets stand for the average value calculated over all possible values of **x**. As a direct consequence of eqn (2), it results from Fourier inversion that the total scattered intensity is proportional to4
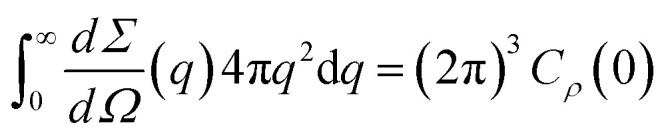
We refer to the left-hand side as *Q*, and its quantitative analysis contributes to the discussion of the scattering by confined microemulsions.

### 3.1 Scattering by two-phase structures

The expressions for the scattering of two-phase structures are well-known.^48,49^ For the purpose of later generalizing them to three-phase structures, it is useful to present them in a way that is mathematically more formal than their usual discussion.

In a two-phase structure, comprising phases A and B with scattering-length densities *ρ*_A_ and *ρ*_B_, the position-dependent scattering-length can be expressed as5
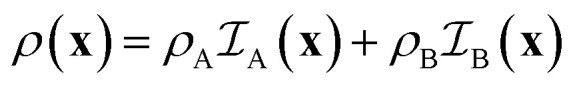
where 
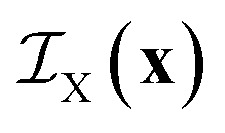
 is the indicator function of phase X, which is equal to 1 if point **x** is inside phase X and to 0 otherwise. Here X can be either A or B. Note that for a two-phase structure 
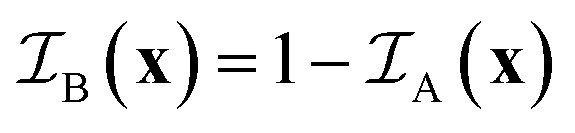
.

In terms of its indicator function, the volume fraction of any phase X is defined as6
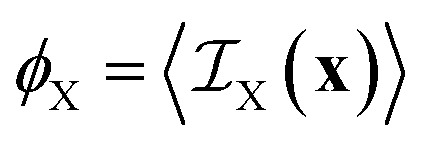
where the brackets 〈〉 stand for the average value calculated over all possible **x**. For further purposes, it is useful to define the correlation function of phase X as7
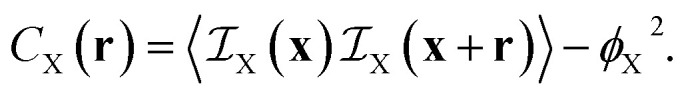
For small values of *r*, the correlation function of any phase takes the asymptotic value^50,51^8
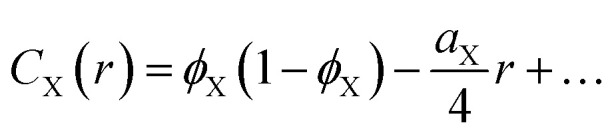
where *a*_X_ is the specific surface area of phase X, and it decreases to *C*_X_(*r*) = 0 for asymptotically larger values of *r*. As an order of magnitude, the distance over which the correlation function decreases to 0 is 4*ϕ*_X_(1 − *ϕ*_X_)/*a*_X_.

In the case of two-phase A/B systems, the correlation function in eqn (7) is identical for phases A and B, namely

9 *C*_A_(*r*) = *C*_B_(*r*) = *ϕ*_A_*ϕ*_B_*Γ*(*r*)

where *Γ*(*r*) is the Debye correlation function, normalized such that *Γ*(0) = 1. From eqn (5), the scattering-length correlation function defined in eqn (3) is equal to

10 *C*_*ρ*_(*r*) = [*ρ*_A_ − *ρ*_B_]^2^*C*_A_(*r*).

It then results from eqn (4) that the total scattered intensity is

11 *Q* = (2π)^3^[*ρ*_A_ − *ρ*_B_]^2^*ϕ*_A_*ϕ*_B_

which is the classical expression of Porod's invariant for two-phase systems.^49^ The scattering cross section is also obtained from eqn (2) as12
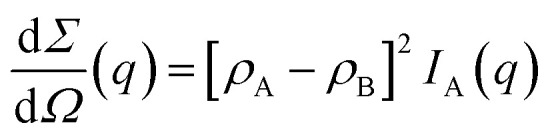
where we use the notation *I*_X_(*q*) for the Fourier transform of *C*_X_(*r*).

For any phase X, the following identity holds13
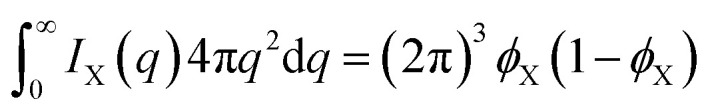
as well as the following asymptotic relation for large *q*14
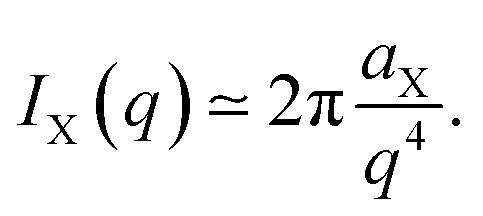
This is known as Porod's law,^49^ and it follows directly from eqn (8)*via* Fourier transformation.^52^

#### 3.1.1 The Teubner–Strey model

A classical model used in the context of scattering by disordered co-continuous structures is provided by the Teubner–Strey structure factor.^24,36^ In addition to the volume fraction *ϕ*_X_, the structure is parameterized through two parameters *d* and *ξ*. With the notations of the present paper, the model takes the form15
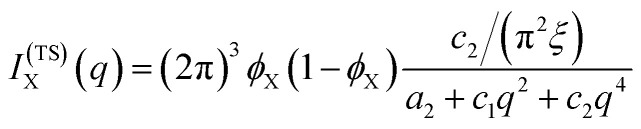
where the constants depend on the model parameters through16
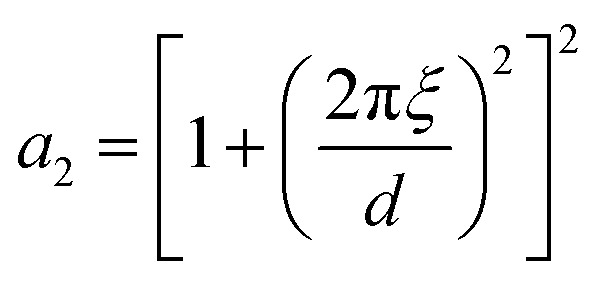
17
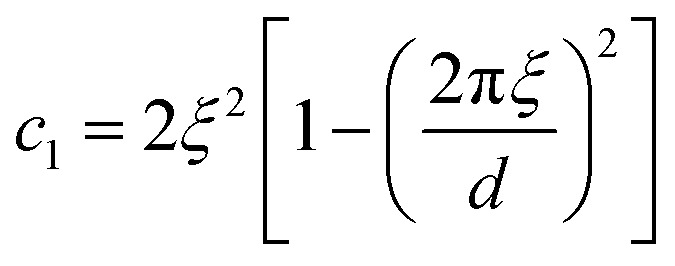
and *c*_2_ = *ξ*^4^.

The function in eqn (15) exhibits a scattering peak for *ξ* > *d*/(2π). In this case, *d* can be interpreted as the spacing between neighboring domains, and *ξ* is the correlation length that controls the sharpness of the scattering peak. The larger *ξ*, the more ordered the structure is.

#### 3.1.2 Clipped Gaussian random field models

As an alternative approach, we also consider clipped Gaussian random field (GRF) models of disordered two-phase structures.^37,53,54^ In that context, a given phase X is modeled as the points of space where a given Gaussian field *W*(**x**) takes values larger than a given threshold *α*. Compared to the Teubner–Strey approach, GRF models ensure that the scattering functions are realizable for any values of the parameters. They also offer more flexibility for generalizing the model to more than two phases.

For our present purpose, a Gaussian field can be conveniently thought of as a superposition of a larger number of waves, namely18
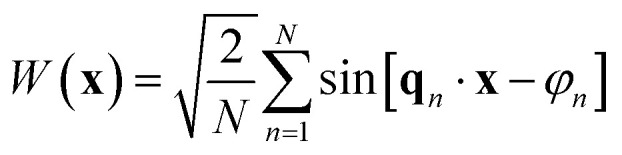
where **q**_*n*_ are random wavevectors drawn from a user-defined distribution function *f*_*W*_(**q**)d*V*_q_, which we refer to as its power spectral density. In the limit of infinitely large *N*, the values of *W*(**x**) are Gaussian distributed with an average equal to 0, and the factor 
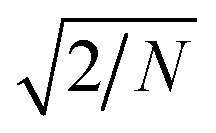
 ensures that the variance is equal to 1.

The field is comprehensively characterized by its spectral density *f*_*W*_(*q*), which in real space is mathematically equivalent to its correlation function

19 *g*_*W*_(*r*) = 〈*W*(**x**)*W*(**x** + **r**)〉

The spectral density *f*_*W*_(*q*) and correlation function *g*_*W*_(*r*) are Fourier transforms of each other. The specific fields we consider in this work have the following correlation function^38,55^20
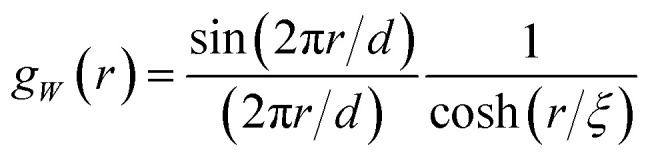
The parameters *d* and *ξ* have similar structural meaning as in the Teubner–Strey model. This specific function has a positive Fourier transform, ensuring the realizability of the Gaussian field. Examples of 2D fields generated with this specific type of correlation function are provided in Fig. 4a and b.

Clipping the Gaussian field at the value *α* boils down to defining the indicator function of phase X as21
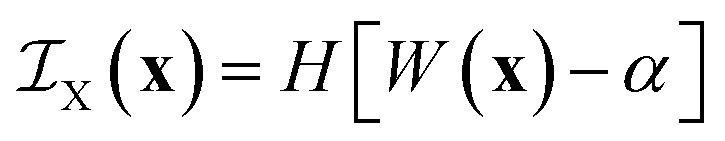
where *H*[] is Heaviside's step function equal to 1 if its argument is positive and to 0 otherwise. The threshold *α* controls the volume fraction *ϕ*_X_. Because the values of *W*(**x**) are Gaussian distributed, the relation is^37,53,54,56^22
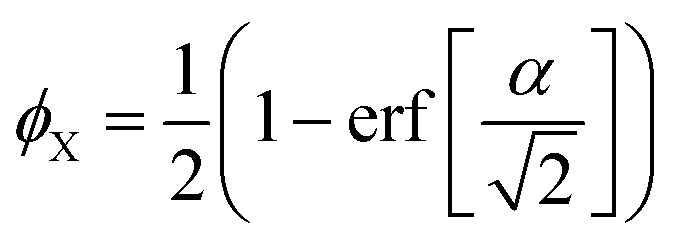
where erf[] is the error function. The correlation function of phase X is calculated as^37,53,54,56^23
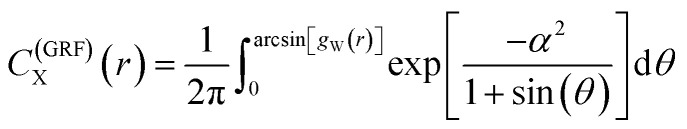
In the specific case where *ϕ*_X_ = 1/2, corresponding to *α* = 0, this relation reduces to *C*^(GRF)^_X_(*r*) = arcsin[*g*_*W*_(*r*)]/(2π).^57,58^

### 3.2 Scattering by three-phase systems

Unlike the systems considered so far, confined microemulsions comprise three phases: the solid phase of the porous glass and the two confined liquids. To analyze the scattering by this type of system, we define the three indicator functions 
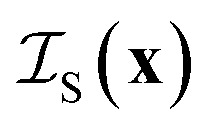
, 
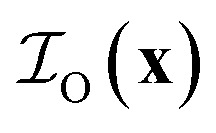
 and 
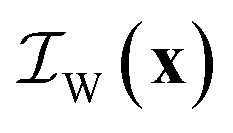
, corresponding to the solid, the *n*-octane and the water, respectively. The space-dependent scattering-length density of the scattering system is now modeled as follows24
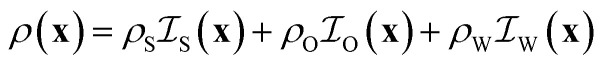
where *ρ*_S_, *ρ*_O_ and *ρ*_W_ are the constant scattering-length densities within each phase.

The scattering-length correlation function is obtained from the general definition in eqn (3), expressed in terms of the six possible self- and cross-correlation functions between three phases. In practice, only three out of these six correlation functions are independent, and the result can be expressed as follows in terms of the self-correlation functions alone^39,59^

25 *Cρ*(*r*) = (*ρ*_S_ − *ρ*_O_)(*ρ*_S_ − *ρ*_W_)*C*_S_(*r*) + (*ρ*_O_ − *ρ*_S_)(*ρ*_O_ − *ρ*_W_)*C*_O_(*r*) + (*ρ*_W_ − *ρ*_O_)(*ρ*_W_ − *ρ*_S_)*C*_W_(*r*)

where *C*_S_(*r*), *C*_O_(*r*) and *C*_W_(*r*) are the correlation functions of the solid, *n*-octane and water.

Evaluating the correlation function *C*_*ρ*_(*r*) in eqn (25) for *r* = 0 provides the following general relation for the integrated scattering intensity of a three-phase system^39^

26 *Q* = (2π)^3^{[*ρ*_S_ − *ρ*_O_]^2^*ϕ*_S_*ϕ*_O_ + [*ρ*_S_ − *ρ*_W_]^2^*ϕ*_S_*ϕ*_W_ + [*ρ*_O_ − *ρ*_W_]^2^*ϕ*_O_*ϕ*_W_}.

This general expression reduces to the classical expression of Porod's invariant in eqn (11) when two phases are indistinguishable, *e.g.* setting *ρ*_O_ = *ρ*_W_ and noting that *ϕ*_O_ + *ϕ*_W_ = 1 − *ϕ*_S_.

#### 3.2.1 Cookie-cutter three-phase model

**Fig. 3:**
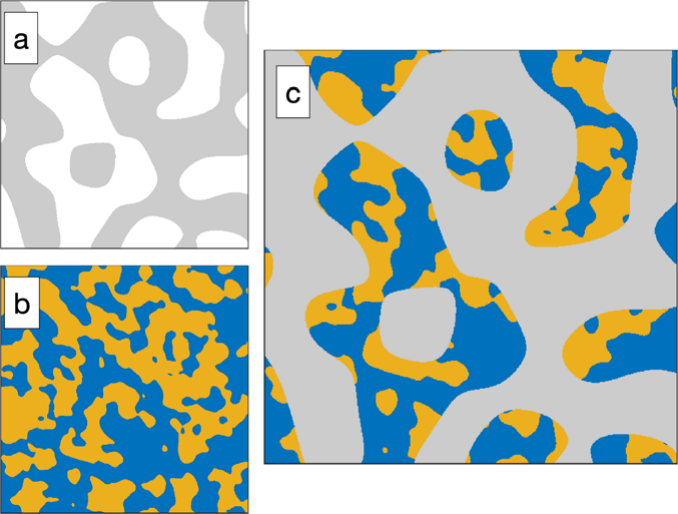
Two-dimensional illustration of the cookie-cutter model of the confined microemulsion, whereby the two-phase porous-glass and microemulsion structures (a and b) are intersected to create the three-phase confined microemulsion structure (c).

The first three-phase model we consider is sketched in Fig. 3. It builds on two independent two-phase models, one for the solid/pore structure (Fig. 3a) and one for the microemulsion (Fig. 3b). We refer to the *n*-octane and water phases of the latter structure as Ō and W̄. They are defined as if they are extended infinitely as in the microemulsion in bulk, and they should not be confused with actual confined phases O and W. The relation between the indicator functions of O/W and Ō/W̄ is27
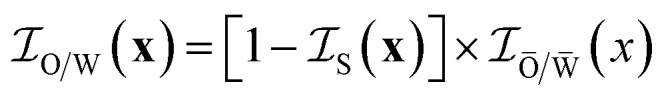
In this equation, the factor 
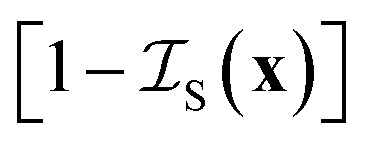
 is the indicator function of the pores. It acts as a mathematical cookie-cutter and restricts the microemulsion structure to the pore space (see Fig. 3).

In eqn (27), the solid and microemulsion structures are assumed to be independent of one another so that the products can be factored out when evaluating averages. In particular, the volume fractions are

28 *ϕ*_O/W_ = (1 − *ϕ*_S_)*ϕ*_Ō/W̄_

where *ϕ*_Ō/W̄_ are the volume fractions of the unrestricted phases, which can also be thought of as the volume fractions within the pores.

Before evaluating the correlation functions *C*_O_(*r*) and *C*_W_(*r*), it is useful to recall that Ō and W̄ make up a two-phase system. Consequently, their correlation functions are equal, in line with eqn (9). We here refer to it as the correlation function of the microemulsion

29 *C*_ME_(*r*) = *C*_Ō_(*r*) = *C*_W̄_(*r*).

With that in mind, the correlation function of the *n*-octane and water phases in the cookie-cutter model is obtained by applying the definition in eqn (7)–(27), which leads to

30 *C*_O/W_(*r*) = (1 − *ϕ*_S_)^2^*C*_ME_(*r*) + *ϕ*^2^_Ō/W̄_*C*_S_(*r*) + *C*_S_(*r*)*C*_ME_(*r*)

The expressions in eqn (30) can, in principle, be used in the general expression of *C*_*ρ*_(*r*) from eqn (25) to calculate the scattering in the Cookie-cutter approximation.

In practice, it is more instructive to make additional assumptions and to consider the two cases where the characteristic length of the solid is either much larger (*d*_P_ ≫ *d*_TS_) or much smaller than that of the microemulsion (*d*_P_ ≪ *d*_TS_). In the former large-pore approximation, the product of correlation functions in eqn (30) can be approximated as

31 *C*_S_(*r*)*C*_ME_(*r*) ≃ *ϕ*_S_(1 − *ϕ*_S_)*C*_ME_(*r*)

This results from noting that *C*_S_(*r*) is almost constant and equal to *C*_S_(0) over distances comparable with the characteristic size of the microemulsion.^55,60^ With that approximation, the scattering-length correlation function in eqn (25) takes the simple form

32 *C*_*ρ*_(*r*) = [*ρ*_S_ − *ρ*_ME_]^2^*C*_S_(*r*) + (1 − *ϕ*_S_)[*ρ*_O_ − *ρ*_W_]^2^*C*_ME_(*r*)

where

33 *ρ*_ME_ = *ϕ*_Ō_*ρ*_O_ + *ϕ*_W̄_*ρ*_W_

is the average scattering-length density of the microemulsion. Alternatively, in the small-pore approximation the product of correlation functions in eqn (30) is approximated as

34 *C*_S_(*r*)*C*_ME_(*r*) ≃ *ϕ*_Ō_*ϕ*_S̄_*C*_S_(*r*)

because *C*_ME_(*r*) is almost constant over the characteristic length of the solid. This eventually leads to

35 *C*_*ρ*_(*r*) = [(1 − *ϕ*_S_)(*ρ*_O_ − *ρ*_W_)]^2^*C*_ME_(*r*) + {*ϕ*_Ō_[*ρ*_S_ − *ρ*_O_]^2^ + *ϕ*_W̄_[*ρ*_S_ − *ρ*_W_]^2^}*C*_S_(*r*)

Eqn (32) and (35) are two limiting cases that are expected to encompass the actual structures of the confined emulsions.

The total scattered intensities in the two regimes are obtained from eqn (4). The results are

36 *Q*^(large pore)^ = (2π)^3^{[*ρ*_S_ − *ρ*_ME_]^2^*ϕ*_S_(1 − *ϕ*_S_) + (1 − *ϕ*_S_)[*ρ*_O_ − *ρ*_W_]^2^*ϕ*_Ō_ϕ_W̄_}

and

37 *Q*^(small pore)^ = (2π)^3^{[(1 − *ϕ*_S_)(*ρ*_O_ − *ρ*_W_)]^2^*ϕ*_Ō_*ϕ*_W̄_ + (*ϕ*_Ō_[*ρ*_S_ − *ρ*_O_]^2^ + *ϕ*_W̄_[*ρ*_S_ − *ρ*_W_]^2^)*ϕ*_S_(1 − *ϕ*_S_)}

The mathematical structure of eqn. (36) and (37) is identical. In both cases, the first term accounts for the scattering by the large-scale structure, where the term between square brackets is the average contrast between the two phases at that scale. In the large-pore situation, there is a contrast between the solid and the whole microemulsion. In the small-pore situation, there is a contrast between the oil-filled and water-filled patches, where only the pore-filling liquid contributes to the contrast. The second term in both eqn (36) and (37) accounts for the scattering by the small-scale structure. In the large-pore situation, this is the microemulsion itself. In the small-pore situation, this is the solid structure contrasted with either *n*-octane or water.

#### 3.2.2 Plurigaussian model

By construction, the cookie-cutter model assumes that the confined liquids and the confining porous solid are independent of one another. This makes the model unsuitable for analyzing possible correlations between the solid phase and the liquid phases. In the specific case where the liquid and solid structures are described with GRF models, the cookie-cutter can be generalized to account for such correlations.

**Fig. 4:**
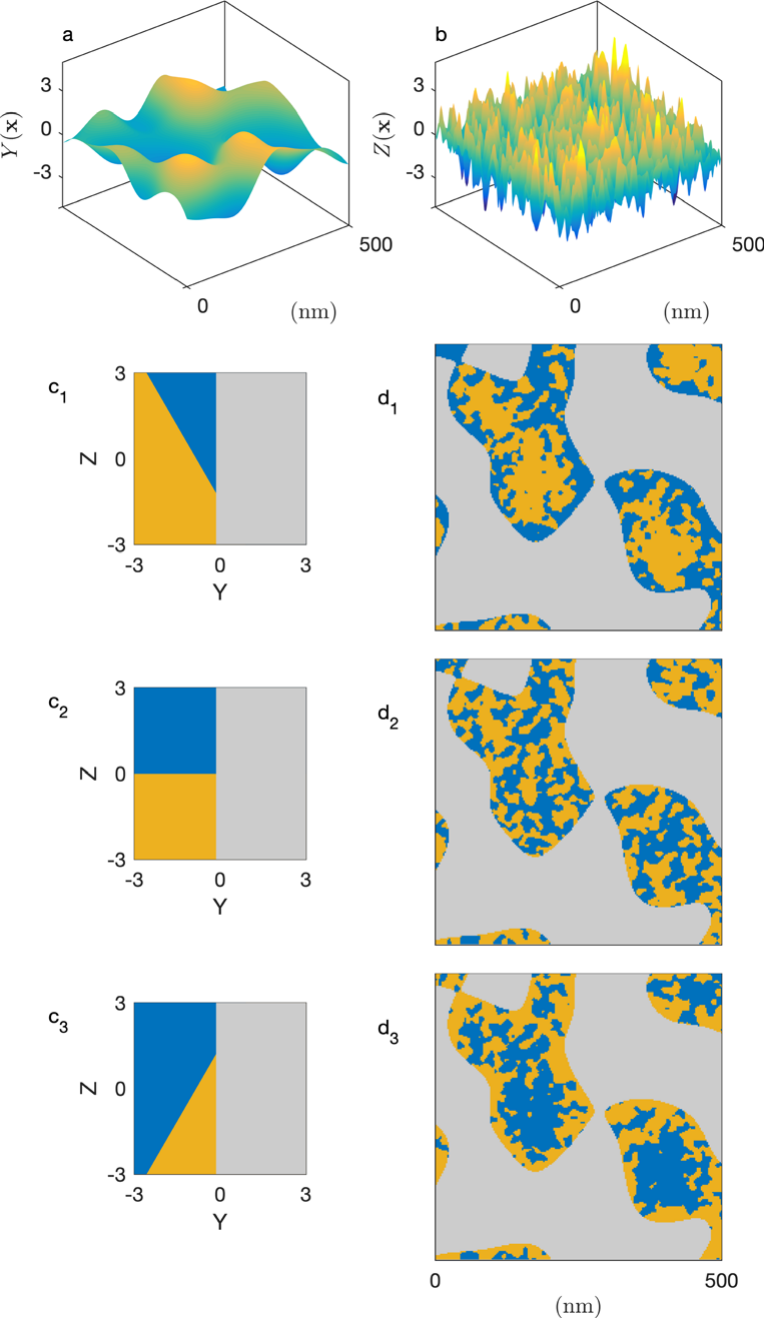
Two-dimensional illustration of the plurigaussian model of confined microemulsion, with the Gaussian fields underlying the solid and microemulsion structures (a and b), the flags of the models (c_1_ to c_3_) and the corresponding structures (d_1_ to d_3_). The solid phase is shown in grey; the water and *n*-octane in blue and yellow, respectively.

In a so-called plurigaussian approach,^38,39,58^ two independent Gaussian fields are considered, say *Y*(**x**) and *Z*(**x**) (see Fig. 4a and b). Structural correlations are introduced through the clipping procedure, by which the real-valued fields are converted to binary all-or-nothing phases. We use here the same criteria as developed in earlier work,^39^ which are illustrated in the flag-like sketches in Fig. 4c_1_–c_3_. These flags represent the specific phase assigned to any point **x**, as a function of the local values of GRF *Y*(**x**) and *Z*(**x**).

In all cases, the solid structure is modeled as a standard clipped GRF, namely as the points of space where *Y*(**x**) > *α*, independently of the value of *Z*(**x**). This is shown in grey in Fig. 4c_1_–c_3_. The particular case of the cookie-cutter model corresponds to Fig. 4c_2_. In that case, the points in the pore space are assigned to the water or oil phase based on the values of *Z*(**x**), independently of *Y*(**x**).

Spatial correlations between the pore-filling liquids and the solid are introduced through oblique boundaries in the model's flag. The case of Fig. 4c_1_ corresponds to a hydrophilic situation, where the pore region close to the surface is enriched in water. The inverse hydrophobic situation is shown in Fig. 4c_3_.

Compared to the cookie-cutter, the plurigaussian model has only one additional parameter, corresponding to the angle *β* between the water/oil and water/solid boundaries in the flag. The correlations turn from hydrophilic to hydrophobic when *β* increase from 0 to π. For any value of *β*, the position of the water/oil interface in the flag space is adjusted to match the volume fractions *ϕ*_Ō_ and *ϕ*_W̄_. All equations of the plurigaussian model necessary to calculate the volume fractions and the correlation functions are provided in Appendix A of our earlier paper.^39^

## 4 Results and discussion

### 4.1 Bicontinuous microemulsion

**Fig. 5:**
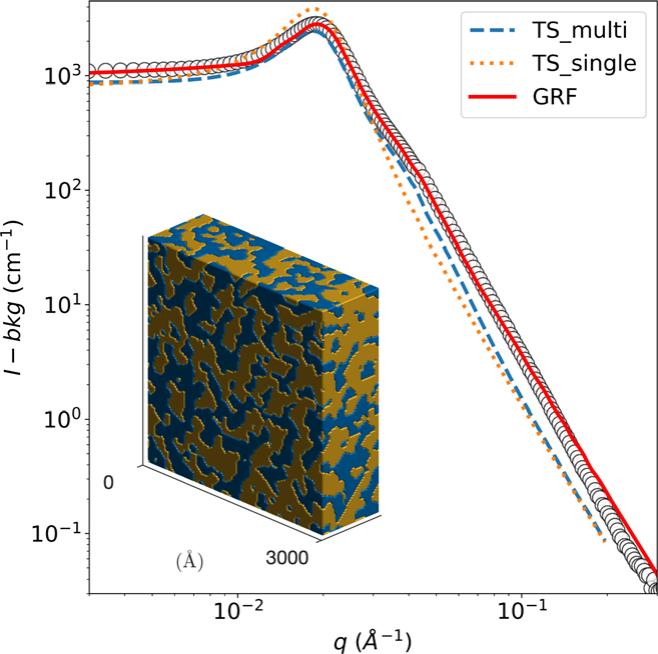
SANS signal of the bicontinuous microemulsion in bulk at 22 °C after subtraction of the incoherent background with the TS fit (orange dotted line), taking multiple scattering into account (blue dashed line). The red line corresponds to the reconstruction, shown in the inset, as a clipped Gaussian random field (GRF).

First, the known phase behavior from the literature was reproduced with the chemicals used. Good agreement was reached, and no further investigation of the bulk phase behavior was necessary. Fig. 5 shows the background-corrected small angle scattering intensity *I*(*q*) of a bicontinuous bulk sample at 22 °C. This SANS signal of the bicontinuous microemulsion in bulk shows the typical broad correlation peak, as shown in Fig. 5. The bicontinuous structure can be described with the Teubner–Strey (TS) model (eqn (15)).^36^

The TS fit describes the broad correlation peak quite well but tends to fail in the high *q*-region. This is caused by a rougher oil–water interface and molecular protrusion.^61^ The bicontinuous microemulsion has a strong scattering signal, often resulting in multiple scattering events.^42,62–64^ This was considered in the analysis by using the semi-analytical convolution method described by Jensen and Barker^65^ and initially developed by Schelten and Schmatz.^66^ The correlation peak is flattened, and a shoulder appears at *q* ≈ 2*q*_max_ due to the multiple scattering; these features are clearly visible in the SANS signal of the bicontinuous microemulsion. The results of the TS fit can be found in Table 1; they are in good agreement with data in the literature.^42^

**Table 1:** Parameters of the Teubner–Strey (TS) and Gaussian random field (GRF) models fitted on the SANS data of the bulk microemulsion (ME) with a surfactant concentration of *γ* = 12.84 wt% and a water-to-octane volume fraction of *ϕ*_W̄_ = 0.5, and for empty porous glasses. The errors associated with *d*_TS_ and *ξ*_TS_ result from averaging over the hydrophilic and hydrophobic CPGs

Sample	*d*_TS_ (nm)	*ξ*_TS_ (nm)	*d*_GRF_ (nm)	*ξ*_GRF_ (nm)
ME	31.6 ± 1.5	15.2 ± 3.2	29.4	9.9
	32.7 ± 3.3^a^	22.2 ± 1.5^a^		
CPG-10	34.3 ± 0.1	18.4 ± 0.1	33.6	16.9
CPG-20	35.9 ± 3.7	26.2 ± 4.8	38.9	20.4
CPG-50	124.3 ± 3.9	51.5. ± 1.5	120.8	59.7
CPG-100	240.6 ± 8.1	126.5 ± 4.1	244.5	115.6

a Corrected for multiple scattering.

The SANS data of the microemulsion was also fitted with the clipped Gaussian Random Field (GRF) model, and the parameters are reported in Table 1. A three-dimensional realization of the model, exhibiting a bicontinuous structure, is displayed in the inset of Fig. 5 where the blue and yellow regions represent the octane and water domains, respectively.

### 4.2 CPG membranes

**Fig. 6:**
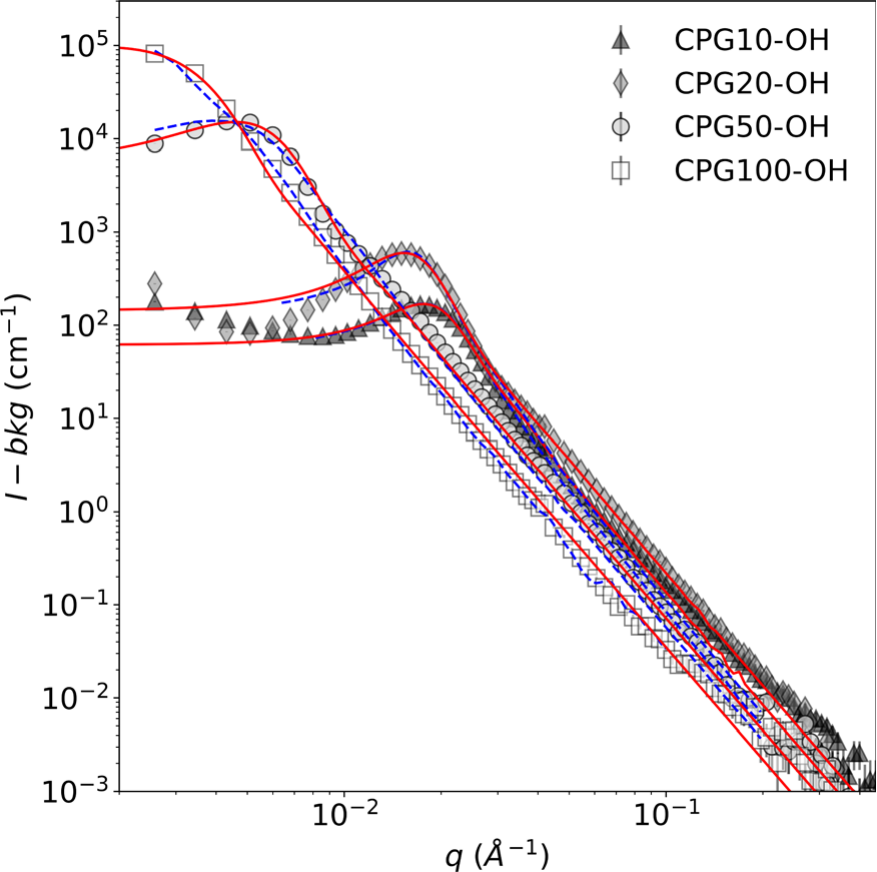
SANS signal of the D_2_O-filled CPG-OH with the TS fit taking multiple scattering into account (blue dashed line) and the GRF fit (red solid line). Only every other data point is displayed for better visibility.

The disordered meso- and macroporous structure of the CPGs is qualitatively similar to the bicontinuous structure of the microemulsion. Hence, the TS fit was used to describe their SANS signal. The most prominent feature in the SANS curves is a broad correlation peak, as reported earlier.^29^Fig. 6 summarizes the SANS data of the hydrophilic CPGs. The position of the peak is shifting to lower *q* with increasing pore diameter. The TS and GRF fits lead to a plateau in the low *q* region, which is not the case for the SANS signal of CPG10 and CPG20. This could result from an incomplete leaching process in the production of the CPG, as reported by Kim *et al.*^67^ Nonetheless, the position and broadness of the peak are well described by both fits.

The SANS signal of the air-filled hydrophilic and hydrophobic CPG are shown in the ESI Fig S3.† The alteration of the surface polarity did not affect the overall shape of the SANS signal, indicating that the pore structure and size remained unchanged. Consequently, the average inter-domain distance obtained from applying the TS fit *d*_TS_ is listed in Table 1. The pore diameter *d*_P_ can be determined from the fits by multiplying *d*_TS_ with the porosity (*ε*). The determined values are in good agreement with the pore diameter obtained from Hg-intrusion measurement *d*_Hg_, as listed in Table 2.

**Table 2:** Pore diameter *d*_Hg_, pore volume *V*_P_, porosity *ε*, which is the fraction of pore volume to total volume, and the specific surface area *A*_S_ obtained from Hg-intrusion and N_2_-adsorption measurements. Pore diameter *d*_P_ obtained from TS fit of the air-filled CPGs by multiplication of the *d*_TS_ (Table 1) with the porosity

CPG	*d*_Hg_ (nm)	*V*_P_ (mL g^−1^)	*ε*	*A*_S_(m^2^ g^−1^)	*d*_P_ (nm)
10	7.00	0.11	0.20	90.9	6.9 ± 0.1
20	16.2	0.21	0.31	60.8	11.3 ± 1.2
50	69.7	0.28	0.38	34.2	46.9 ± 1.5
100	131	0.36	0.44	21.1	106.8 ± 3.6

**Fig. 7:**
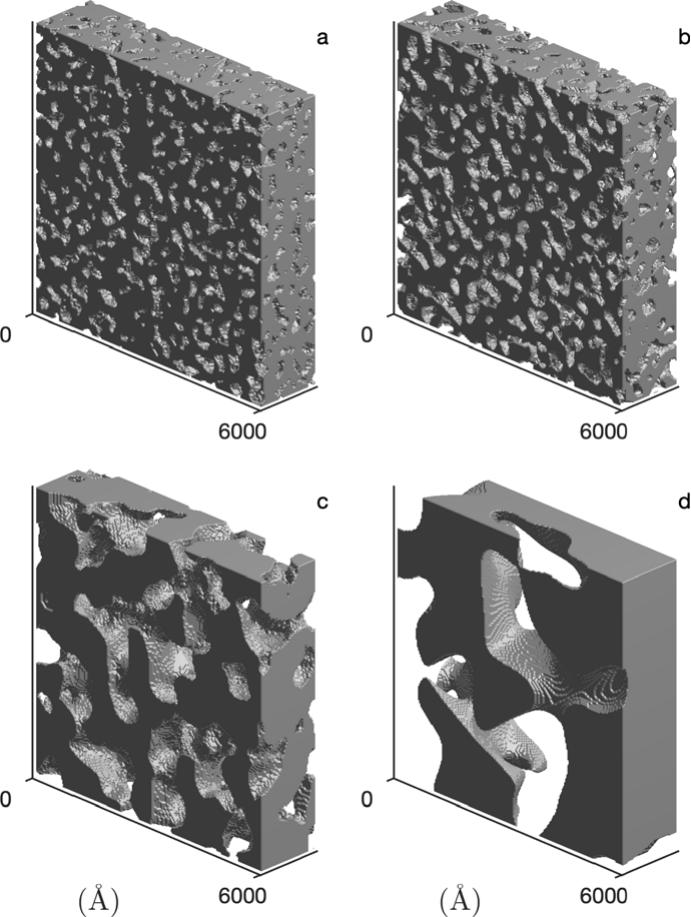
The reconstructed nanostructure of the CPG, (a–d) for CPG10, CPG20, CPG50 and CPG100, respectively. The reconstructions correspond to the GRF model of the SANS signal of the D_2_O-filled hydrophilic CPG shown in Fig. 6.

Application of the GRF fit to the SANS data of the CPG allows for the reconstruction of the disordered porous matrix, as shown in Fig. 7. The pore wall material and the empty pore volume are depicted in dark grey and white, respectively.

### 4.3 Confined microemulsions

**Fig. 8:**
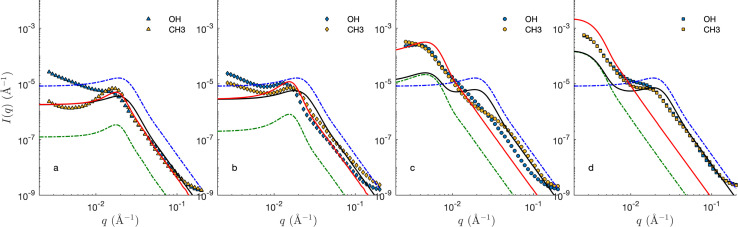
SANS signal of the confined microemulsion in CPG10 (a), CPG20 (b), CPG50 (c), and CPG100 (d), with hydrophilic (blue) and hydrophobic (orange) surfaces. The dashed blue line is the fitted scattering from the microemulsion in bulk (GRF model), and the dashed green lines are the fitted CPG scaled for silica-microemulsion contrast (GRF model). The solid black and red lines are cookie-cutter models of bicontinuous and phase-separated emulsions.

Fig. 8 shows the SANS of the CPGs filled with the microemulsion (ME). For comparison, the scattering of the bulk (unconfined) microemulsion is plotted on the same graphs (dashed blue), as well as that of the empty CPGs rescaled for the effective contrast between the silica and the average microemulsion. For the largest pores, the contributions of the solid and ME can be qualitatively identified (see Fig. 8d). For the smaller pores, however, the correlation peaks of the CPG and ME are almost at the same *q*-position, making it difficult to discriminate them (Fig. 8a).

#### 4.3.1 Analysis of the scattering invariant *Q*

Scattering insights into complex three-phase systems can be obtained by considering the invariant *Q*. Because the invariant depends only on the volume fractions of the scattering phases and on their scattering-length densities, the analysis is independent of any specific structural model.^30,39,68^

**Fig. 9:**
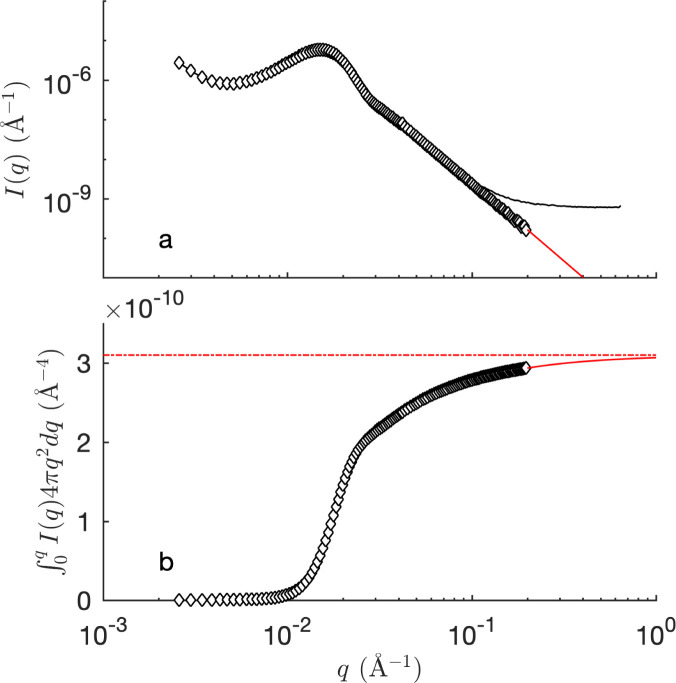
Evaluation of the integrated intensity *Q* from the experimental SANS signal (black line in (a)), based background subtraction to enforce Porod scattering (red line). The dashed red line in (b) shows the value of *Q* after extrapolation. The shown data is that of hydrophilic CPG20 filled with D_2_O.

The invariant is determined experimentally following eqn (4), by integrating the SANS intensity.^30,68^ The procedure is illustrated in Fig. 9, and involves subtracting first a background contribution followed by an extrapolation using *q*^−4^ Porod's law. The so-obtained values of *Q* for the air- and D_2_O-filled CPGs are plotted systematically against the solid volume fraction in the ESI Fig. S4.† Comparison with the classical two-phase result in eqn (11) reveals an average relative error of 15%. This value is used hereafter as a confidence interval for *Q*. Deviations might result from adsorbed H_2_O on the silica walls, influencing the contrast. In the case of the large-pore samples CPG50 and CPG100, the SANS intensity might also be affected by neutron refraction.

**Fig. 10:**
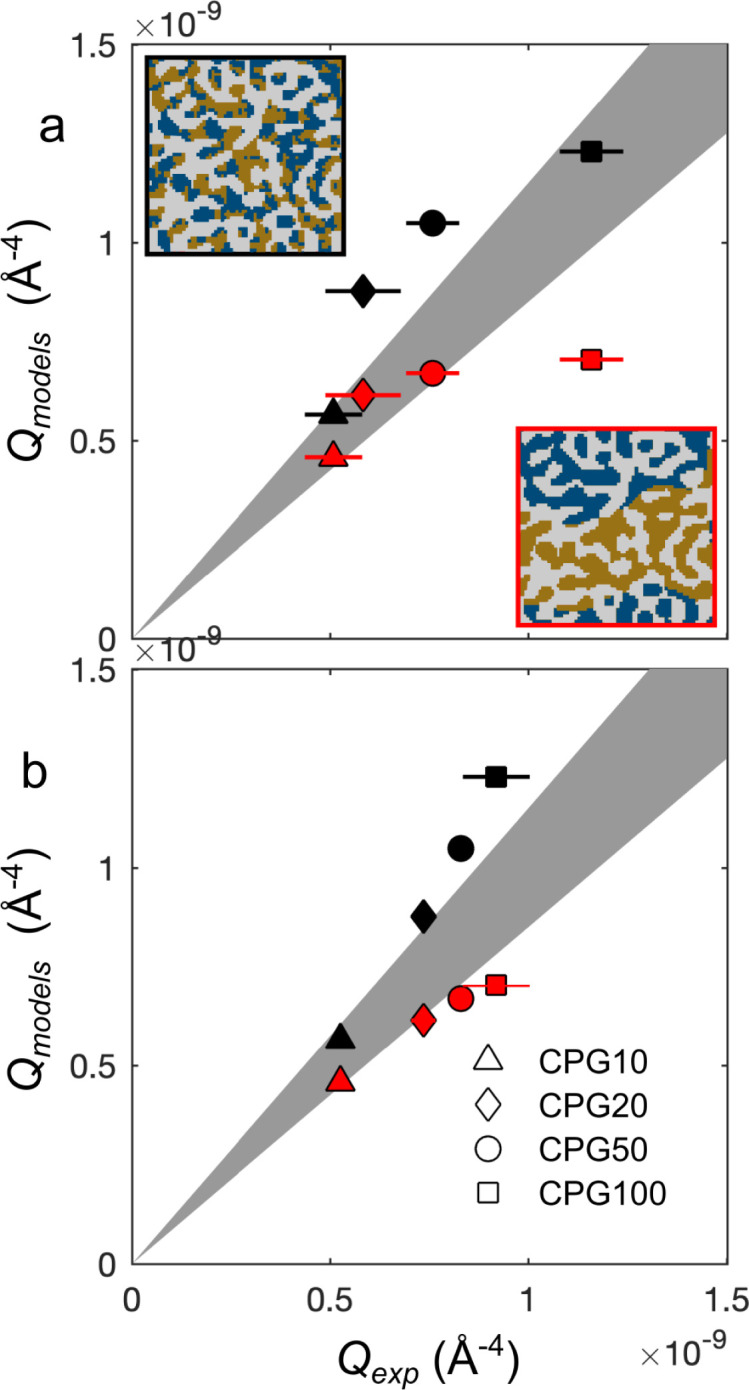
Comparison of the modeled *versus* experimental values of *Q*, black symbols assume bicontinuous structure, red symbols assume phase separation for the hydrophilic CPG-OH (a) and the hydrophobic CPG-CH_3_ (b). The grey area highlights the confidence interval. The insets illustrate the two modeled phase behaviors of the microemulsion inside the pores, the upper one shows a bicontinuous microemulsion and the lower one a phase-separated microemulsion inside the pores.

Fig. 10 plots the integrated intensities of the CPG-confined microemulsions, and compares them with two general models. In case the microemulsion in the pores would remain bicontinuous, the scattering signal would originate from pores filled with oil- and water domains, results in a three-phase system as depicted in the inset Fig. 10. In this case, the values of *Q* should be given by the general three-phase expression in eqn (26) as a function of the known volume fractions and scattering-length densities (black symbols in Fig. 10). Alternatively, we also consider the extreme case of macroscopic phase separation of the microemulsion as visualized in the bottom inset in Fig. 10. In that case, a significant fraction of the scattering by the confined microemulsion would occurs outside of the measured *q* range, and the detected scattering signal would originate from either a water-domain-filled pore or an oil-domain-filled pore. In this scenario the invariant *Q* would be expressed through eqn (37), and these values are plotted as red symbols in Fig. 10. In the same figure, the gray area is the ±15% confidence interval.

For the hydrophilic CPG100, it is clear that modeling the bicontinuous structure leads to good agreement. However, the modeled values for smaller pores exceed the experimentally determined values and fall outside the confidence interval. For CPG10, *Q*_models_ for both scenarios fall within the confidence interval. This might be caused by the small contribution of the microemulsion to the scattering signal. Since the porosity of this sample is only 20%, meaning that the scattering signal mainly arises from the porous solid. Additionally, the pore diameter (*d*_P_) is 7 nm, much smaller than the size of one oil or water domain of the microemulsion. If a bicontinuous structure is assumed, this would still lead to a scattering from either a water-filled or an oil-filled pore. So, it is difficult to distinguish the two scenarios for very small pores. For the tested hydrophilic samples, it can be concluded that a phase separation of the microemulsion is likely to occur if the *d*_P_ is less than 45 nm. In the case of the macroporous CPG100, no phase alteration is indicated, and the microemulsion structure remains bicontinuous.

The case for the hydrophobic CPG-CH_3_ is not as clear compared to CPG-OH. The *Q*_models_-values for the confined microemulsion inside the hydrophobic pores are shown in Fig. 10b). For these samples, the bicontinuous structure results in values above the confidence interval, while the phase-separated case results in values below. This may suggest that for the confined microemulsion, both scenarios are partially accurate, and a portion of the microemulsion remains bicontinuous while the other portion is phase-separated. This might be caused by the difference in the adsorption of the surfactant onto the CPG surfaces. Neutron reflectometry experiments might shed light on the near-surface structure of the microemulsions. This was done previously with bicontinuous microemulsions based on sugar surfactants.^12,13^ Only slight differences in the near-surface structure close to hydrophilic and hydrophobic surfaces were observed. These surfactants are known to adsorb barely on silica in contrast to the used surfactant in this work.^69^

These results show that the structure of the confined microemulsion is influenced by the pore diameter as well as the composition of the pore walls. The latter indicates a difference in the interactions of the microemulsion with a hydrophilic and a hydrophobic surface.

#### 4.3.2 Gaussian and Plurigaussian modelling

The two general scenarios considered in Fig. 10 are here compared to the SANS of the confined microemulsions in Fig. 8, assuming specific structural models.

The red lines in Fig. 8 are calculated based on phase separation. Because phase separation is equivalent to assuming that the oil/water domains are much larger than that of the CPG, its scattering can be modelled using the small-pore approximation introduced in eqn (35). Furthermore, if the separation is macroscopic, oil/water structure contributes only to the forward scattering and the first term in eqn (35) can be ignored. The scattering is then proportional to that of the CPG. The so-calculated scattering – assuming the GRF model for the CPGs – captures reasonably the SANS of CPG10 and CPG20. Upwards deviation are observed at small *q*, which hints at the fact that phase separation is not quite macroscopic. In the case of the CPGs with largest pores, the differences between the calculated and experimental scattering, rule out phase separation.

The scattering of the ME in the CPG with the largest pore can be captured with the large-pore approximation model introduced in eqn (32). This results in additive contributions of the solid and microemulsion to the scattering, and the so-calculated patterns are plotted as black lines in Fig. 8. The agreement of these calculations with the SANS of the small-pore CPGs seems to be reasonable, but this is a coincidence resulting from the fact that characteristic sizes of the ME and CPGs are similar. In reality, the very assumption of the large-pore approximation do not apply to CPG20. In the case of CPG100, the two steps in the SANS are accounted for by the model, but the agreement is mostly qualitative at small *q*.

**Fig. 11:**
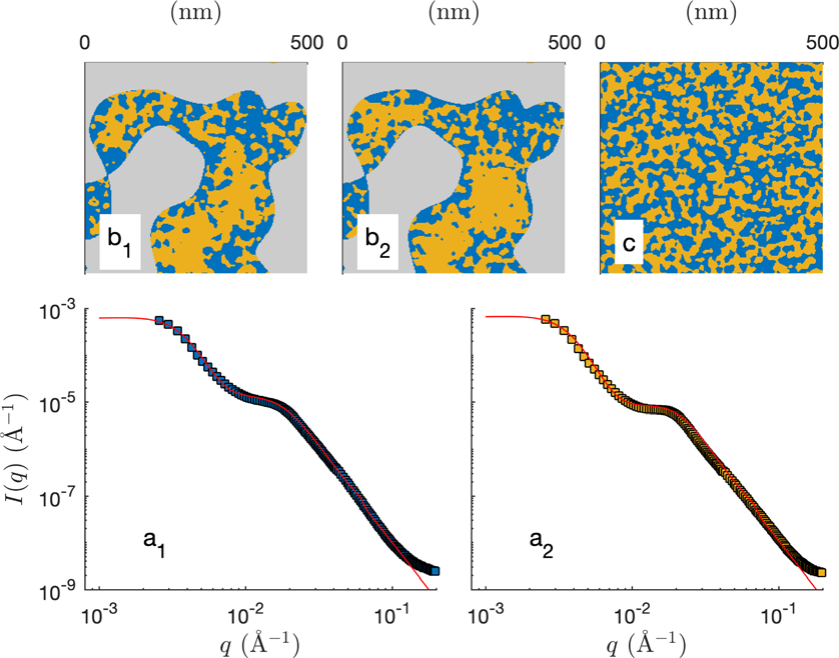
Plurigaussian fits of the confined microemulsions in CPG100, with OH- (a_1_) or CH_3_-coated (a_2_) surfaces. The dots are the data (same as Fig. 8d), and the solid lines are the plurigaussian fits. Two-dimensional realizations of the models with the fitted parameters are shown in b_1_ and b_2_. A two-dimensional realization of the unconfined microemulsion is shown in c for comparison.

The two models presented in Fig. 8 are not fitted to the data, as they are not based on any adjusted parameter. Moreover, they are both based on the general cookie-cutting construction of Fig. 3, which cannot account for solid/microemulsion correlations. We now consider a more sophisticated plurigaussian model to fit the SANS of the microemulsion in macroporous CPG100 as illustrated in Fig. 11. For the fits, the characteristics of the CPGs are kept constant, but the parameters of the confined microemulsion *ξ* and *d* are adjusted as well as the angle *β*, characterizing the correlation between the solid and microemulsion structures (see Fig. 4). This latter parameter can also be thought of as an average contact angle between the water/oil and water/solid phases, and the fitted value is *β* ≃ 48°. The plurigaussian fits reveal that water is enriched at the pore walls while *n*-octane accumulates in the center of the pore, as depicted in Fig. 11b_1_ and b_2_. The fits and the corresponding reconstruction of the confined microemulsion inside the hydrophilic and hydrophobic pores are very similar. This counter-intuitive situation might be caused by surfactant adsorption on the pore walls, allowing water enrichment even at the hydrophobic pore walls.

#### 4.3.3 Implications for the phase behavior inside porous matrices

**Fig. 12:**
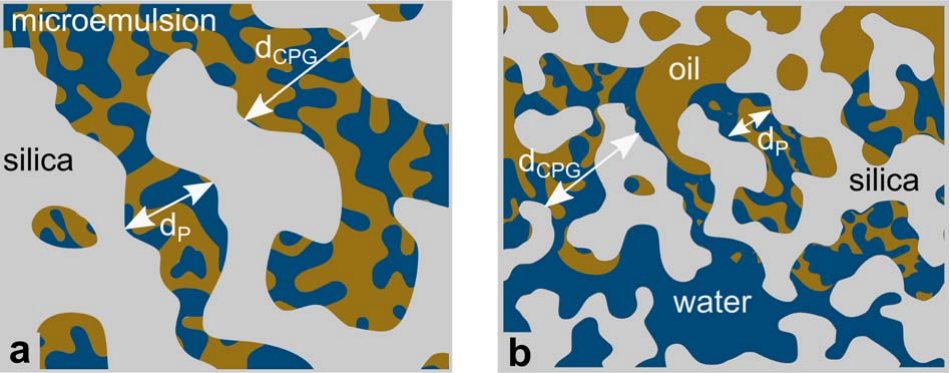
Possible phase behavior of the microemulsion in large pores (a) and small pores (b).

The evaluation of the scattering data suggests that the phase structure of the microemulsion inside the pores of CPG20 and CPG10 undergoes a change, as visualized in Fig. 12. At a given temperature and composition, the phase structure is thermodynamically fixed. Since the temperature of the entire system is constant and the volumes of C_10_E_4_–H_2_O–*n*-octane within the overall system of microemulsion and pores remain the same, a reduction of the volume of at least one component of the microemulsion must take place to change the phase structure at the given temperature. As can be seen in the phase diagrams of Fig. 1, decreasing the surfactant concentration shifts the composition towards lower values of the parameter *γ*, and eventually, the three-phase region is reached, and excess oil and water occurs due to the loss of surfactant in the microemulsion. If the volume of one of the bulk phases is reduced, the oil-to-water ratio is altered, and the bicontinuous structure becomes asymmetric. We assume the shift of *γ* to be responsible for the observed structural changes. Non-ionic surfactants of the (C_*i*_E_*j*_) class are known to adsorb strongly on silica surfaces.^69^

The surface area from the porous samples used in our experiments can be determined by multiplication of the specific surface area *A*_S_ from Table 2 with the weight of the used CPGs. This surface area varies from 15 m^2^ for CPG10 to 3.6 m^2^ for CPG100. Considering an area per surfactant molecule of 54 Å^2^,^42^ 3 × 10^19^ molecules for CPG10 and 7 × 10^18^ for CPG100 would be required to fully cover the entire surface of the porous solid. As the volume of the used microemulsion contains a few 10^18^ to 10^19^ surfactant molecules, the microemulsion would cease to exist, leaving only phase-separated oil and water. This ratio of internal surface area to surfactant molecules in this work is much higher than in performed neutron reflectometry experiments,^10,13^ which makes a comparison more difficult. The reported lamellar ordering^10^ was not observed in the presented SANS data.

Since we clearly observe contributions of bicontinuous structure at least from the confined microemulsion in CPG100, we can rule out that all of the surfactant of the microemulsion is adsorbed at the pore walls. More likely, a fraction of surfactant molecules will adsorb at the pore walls and shift the composition mainly towards lower values of *γ* without significant changes in the oil-to-water ratio. At least for CPG10 and CPG20, a minor shift of *q*_max_ towards lower values was observed. Such a shift towards lower *γ* is accompanied by an increase of *d*_TS_. If one takes into account that reducing *γ* by 0.5% leads to a change of the inter-domain distance Δ*d*_TS_ of 1 nm, then a reduction of *γ* by 7% can be estimated from the shift in *q*_max_ in Fig. 8. At constant temperature, the single-phase bicontinuous structure of the microemulsion changes towards a three-phasic structure with a bicontinuous middle phase inside the porous material of CPG20 and CPG10. For a sufficiently large volume, some pores are filled with a bicontinuous phase while others are filled with pure bulk phases, creating the impression of partial phase separation. Since all experiments were performed at a constant temperature, a possible shift of the phase boundaries due to the confinement reported for binary systems^33^ was not observed.

These considerations lead to the hypothesis that the geometric pore size (*d*_P_) is not the decisive parameter but the inner surface area available for surfactant adsorption in the material. This size is determined by the pore diameter and the porosity *ε*. However, this effect differs from the influence of the pore size on the critical fluctuations in binary mixtures as soon as the characteristic length in the fluctuating system is comparable with the pore size.

## 5 Conclusion

This work investigates the behavior of a bicontinuous microemulsion confined in a porous material compared to other liquid systems like binary mixtures. SANS experiments revealed that confinement effects depend on the pore diameter, the porosity of the confining matrix, and its surface composition.

Two potential scenarios were considered as extreme cases: first, where the confined microemulsion remains bicontinuous, and second, where the oil and water domains are separated. Structure-independent modeling based on the scattering invariant *Q* suggests that in hydrophilic pores the microemulsion is more likely to remain bicontinuous in larger pores, while smaller pores favor phase separation. In hydrophobic pores, both scenarios may be partially true. The deviation of the confined microemulsion inside the hydrophilic and hydrophobic pores shows that the interaction with the surface depends on its polarity. For the confined microemulsion inside the largest pores (CPG100), the results of the plurigaussian fits indicate that for both surface polarities, there is an enrichment of water at the pore walls and *n*-octane in the pore center.

It can be reasonably concluded that the most probable scenario is the adsorption of surfactant molecules at the pore walls, which would result in a shift in the microemulsion composition. This leads to a shift in the fish-type phase diagram towards lower surfactant concentrations and towards the three-phase region at constant temperature. That could be indistinguishable from a partial phase separation.

Neutron reflectometry experiments on both surfaces are necessary for a deeper understanding. Self-diffusion NMR and neutron spin echo experiments can further investigate the phase behavior of the microemulsion inside the pores. The observed behavior is relevant to all systems containing a bicontinuous microemulsion or a disordered porous material, especially for applications in soil remediation or enhanced oil recovery.

## Data availability

Proposal number of the SANS measurements at the ILL, Grenoble, is 9-10-1736 (10.5291/ILL-DATA. 9-10-1736). Proposal number of the SANS measurements at ANSTO, Sydney, is 16582.

## Author contributions

The manuscript was written by MD, CG, and SW. MD, RH, TO, TJS, and SW performed the SANS experiments. SP and KW are the instrument responsibles for D22 and Quokka, respectively. The TS fits were performed by MD. The three-phase models were developed and fitted by CG. Data were evaluated and discussed by MD, CG, TH, and SW. PS performed the determination of the BET surface area. The surfactant adsorption was investigated by MD.

## Conflicts of interest

There are no conflicts to declare.

## Supplementary Material

RA-014-D4RA04090B-s001
