# Supplementary material for: Confinement induced change of microemulsion phase structure in controlled pore glass (CPG) monoliths
Source: RSC Adv. 2024 Sep 4;14(39):28272–84. doi: 10.1039/d4ra04090b (PMC11372560; doi:10.1039/d4ra04090b)
Supplement: RA-014-D4RA04090B-s001 [file RA-014-D4RA04090B-s001.pdf]

Supplementary Information (SI) for RSC Advances.
This journal is © The Royal Society of Chemistry 2024

Supporting Information

Confinement Induced Change of Microemulsion Phase Structure in Controlled Pore Glass (CPG) Monoliths

Margarethe Dahl,[a] Cedric J. Gommes,[b] René Haverkamp, [c] Kathleen Wood, [d] Sylvain Prévost, [e] Pierre Schröer, [a] Tomáš Omasta, [a] Tim Julian Stank, [c] Thomas Hellweg, [c] and Stefan Wellert [a]

[a] *Department of Chemistry, Technische Universität Berlin, Straße des 17. Juni 124, 10623 Berlin, Germany. E-mail: s.wellert@tu-berlin.de*
[b] *Department of Chemical Engineering, University of Liège B6 A, 3 allée du 6 aout, B-4000 Liège, Belgium.*
[c] *Department of Physical and Biophysical Chemistry, University of Bielefeld, Universitätsstraße 25, 33615 Bielefeld, Germany.*
[d] *Australian Nuclear and Technology Organisation, New Illawara Rd, Lucas Heights, NSW 2234, Australia*
[e] *Institute Laue-Langevin, 71 avenue des Martyrs, F-38042 Grenoble, France.*

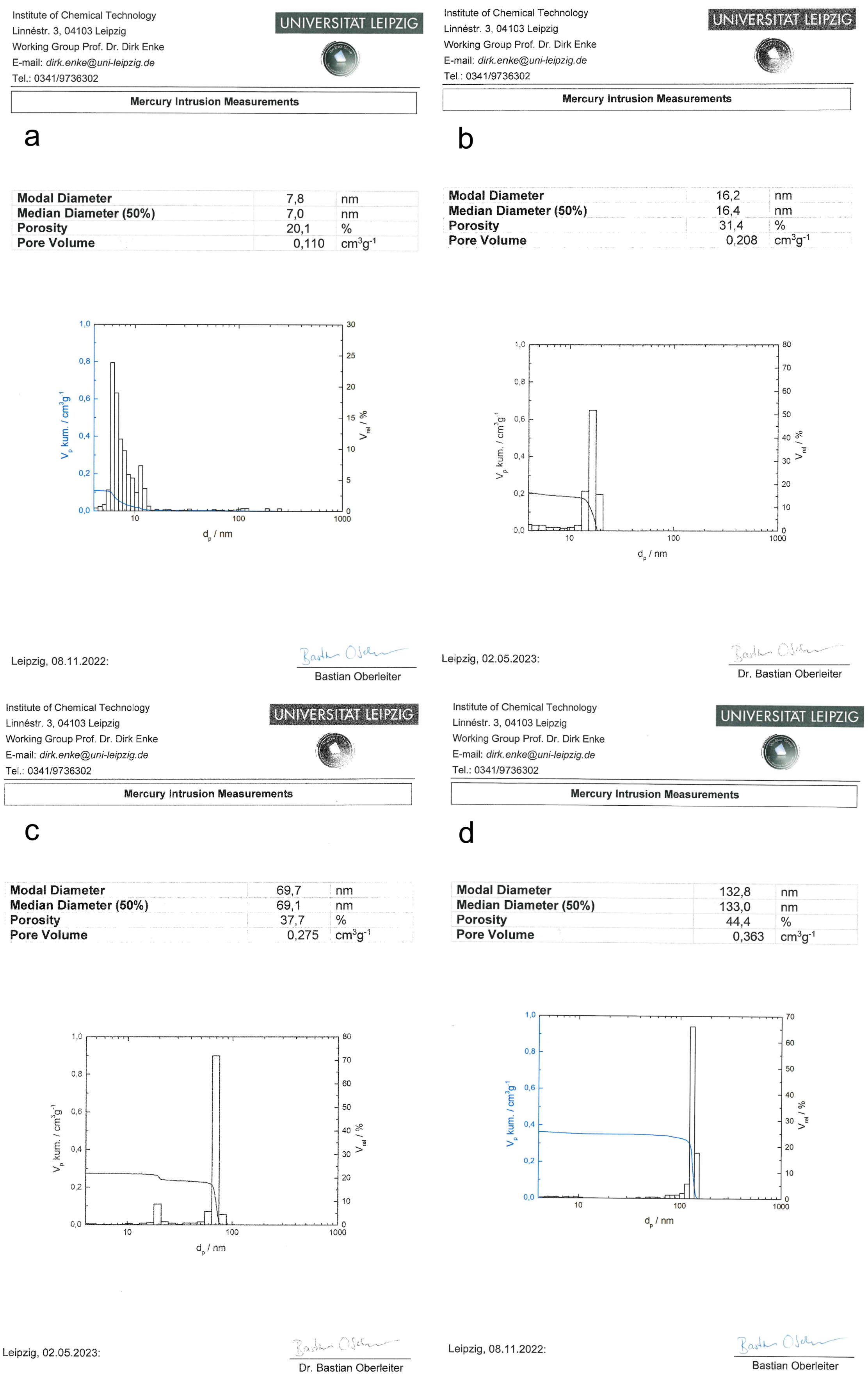

Institute of Chemical Technology
Linnéstr. 3, 04103 Leipzig
Working Group Prof. Dr. Dirk Enke
E-mail: *dirk.enke@uni-leipzig.de*
Tel.: 0341/9736302

UNIVERSITÄT LEIPZIG

Mercury Intrusion Measurements

a

**Modal Diameter**	7,8	nm
**Median Diameter (50%)**	7,0	nm
**Porosity**	20,1	%
**Pore Volume**	0,110	cm^3g^{-1}

Leipzig, 08.11.2022:

Bastian Oberleiter

Institute of Chemical Technology
Linnéstr. 3, 04103 Leipzig
Working Group Prof. Dr. Dirk Enke
E-mail: *dirk.enke@uni-leipzig.de*
Tel.: 0341/9736302

UNIVERSITÄT LEIPZIG

Mercury Intrusion Measurements

b

**Modal Diameter**	16,2	nm
**Median Diameter (50%)**	16,4	nm
**Porosity**	31,4	%
**Pore Volume**	0,208	cm^3g^{-1}

Leipzig, 02.05.2023:

Dr. Bastian Oberleiter

Institute of Chemical Technology
Linnéstr. 3, 04103 Leipzig
Working Group Prof. Dr. Dirk Enke
E-mail: *dirk.enke@uni-leipzig.de*
Tel.: 0341/9736302

UNIVERSITÄT LEIPZIG

Mercury Intrusion Measurements

c

**Modal Diameter**	69,7	nm
**Median Diameter (50%)**	69,1	nm
**Porosity**	37,7	%
**Pore Volume**	0,275	cm^3g^{-1}

Leipzig, 02.05.2023:

Dr. Bastian Oberleiter

Institute of Chemical Technology
Linnéstr. 3, 04103 Leipzig
Working Group Prof. Dr. Dirk Enke
E-mail: *dirk.enke@uni-leipzig.de*
Tel.: 0341/9736302

UNIVERSITÄT LEIPZIG

Mercury Intrusion Measurements

d

**Modal Diameter**	132,8	nm
**Median Diameter (50%)**	133,0	nm
**Porosity**	44,4	%
**Pore Volume**	0,363	cm^3g^{-1}

Leipzig, 08.11.2022:

Bastian Oberleiter

Fig. S 1 Hg-intrusion certificates of the porous membranes CPG10, CPG20, CPG50 and CPG100 (a – d) from the supplier.

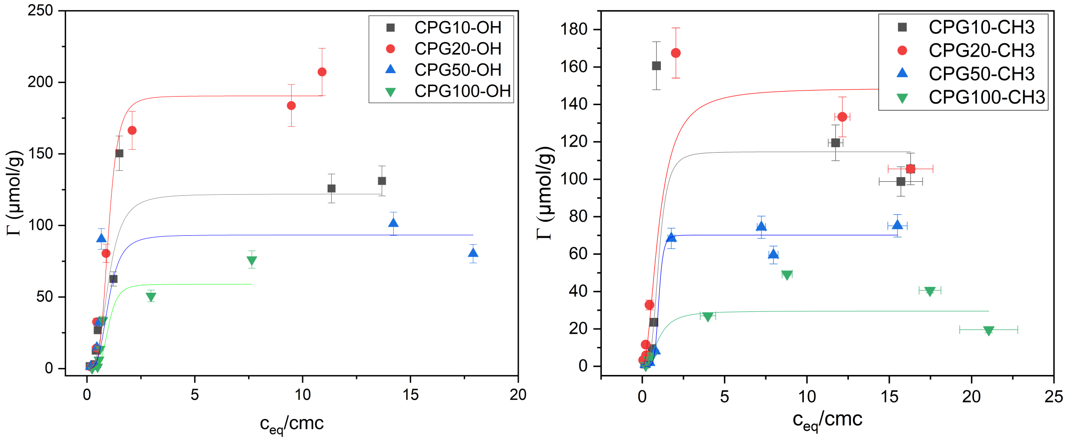

Fig. S 2 Adsorption Isotherms of the $C_{10}E_4$ surfactant onto the hydrophilic CPG (left) and hydrophobic CPG (right) with the fit of the Gu-Zhu model.

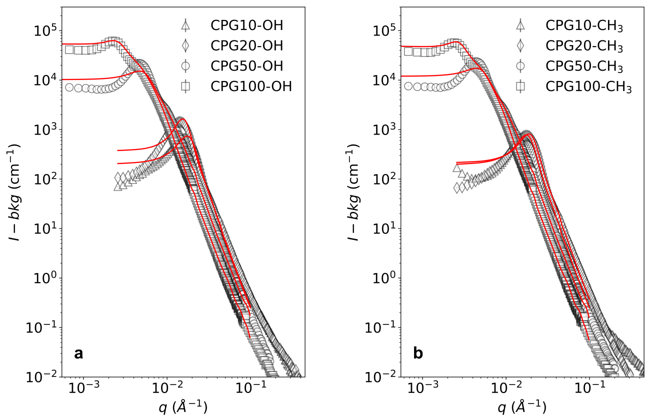

Fig. S 3 a) SANS signal of the air-filled hydrophilic CPG-OH and b) hydrophobic CPG-CH_3 after subtraction of the incoherent background with the TS fit taking multiple scattering into account (red line).

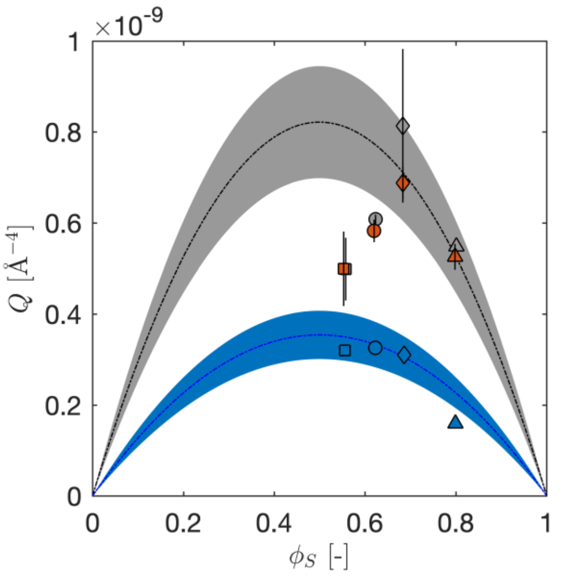

Fig. S 4 Integrated total scattering intensity Q_{exp} versus solid volume fraction Φ_S. The blue symbols represent the D_2O-filled hydrophilic samples. The air-filled CPG-CH_3 is shown as red symbols and the air-filled CPG-OH is shown as gray symbols. The error bars are from experiments on different instruments.
